# Simultaneous acquisition of dynamic PET-MRI: arterial input function using DSC-MRI and [18F]-FET

**DOI:** 10.1186/2197-7364-2-S1-A70

**Published:** 2015-05-18

**Authors:** Liliana Caldeira, Seong Dae Yun, Nuno da Silva, Christian Filss, Juergen Scheins, Lutz Telmann, Hans Herzog, Jon Shah

**Affiliations:** Institute of Neuroscience and Medicine – 4, Forschungszentrum Juelich GmbH, Germany

This work focuses on the study of simultaneous dynamic MR-PET acquisition in brain tumour patients. MR-based perfusion-weighted imaging (PWI) and PET [18F]-FET are dynamic methods, which allow to evaluate tumour metabolism in a quantitative way. In both methods, arterial input function (AIF) is necessary for quantification. However, the AIF estimation is a challenging task. In this work, we explore the possibilities to combine dynamic MR and PET AIF.

## Materials/Methods

Data of 11 brain tumour patients was acquired in a hybrid 3TMR-BrainPET (Siemens). The PET acquisition took one hour while several MRI sequences were simultaneously acquired, including DSC-MRI (Dynamic Susceptibility Contrast MRI) and MPRAGE. Two DSC-MRI sequences for PWI were investigated: 1) echo planar imaging (EPI) and 2) EPI Keyhole (EPIK). For three patients venous blood samples were available at later times. For DSC-MRI AIF estimation an automatic in-house algorithm was used and for PET AIF estimation an automatic segmentation of the carotid arteries in MPRAGE images was used. Furthermore, several anatomical-based partial volume correction (PVC) methods were applied to images using the carotid artery segmentation.

## Results

The gamma fitting is useful in MR studies where the first pass of the bolus is used for quantification and can also aid the estimation of the peak of the PET AIF due to the similar peak shape. For the later PET frames, a three exponential model provided a good fitting. The iterative Yang PVC provided results closer to the venous blood samples than the other PVC methods.

## Conclusion

PET quantification can be improved by using MR data for: automatic carotid segmentation, partial volume correction and estimation of AIF using dynamic MRI. Furthermore, the estimation of MR AIF using EPIK instead of EPI was more robust and consequently PET AIF.

